# Occurrence of *Enterocytozoon bieneusi* in Donkeys (*Equus asinus*) in China: A Public Health Concern

**DOI:** 10.3389/fmicb.2017.00565

**Published:** 2017-03-31

**Authors:** Dong-Mei Yue, Jian-Gang Ma, Fa-Cai Li, Jun-Ling Hou, Wen-Bin Zheng, Quan Zhao, Xiao-Xuan Zhang, Xing-Quan Zhu

**Affiliations:** ^1^State Key Laboratory of Veterinary Etiological Biology, Key Laboratory of Veterinary Parasitology of Gansu Province, Lanzhou Veterinary Research Institute, Chinese Academy of Agricultural SciencesLanzhou, China; ^2^College of Animal Science and Technology, Jilin Agricultural UniversityChangchun, China; ^3^College of Animal Science and Technology, Changchun Sci-Tech UniversityShuangyang, China

**Keywords:** *Enterocytozoon bieneusi*, Microsporidia, donkey, zoonotic disease, Internal transcribed spacer (ITS)

## Abstract

*Enterocytozoon bieneusi* is an important zoonotic parasite. It can infect virtually all animal species and has a global distribution. However, the prevalence of *E. bieneusi* in donkeys (*Equus asinus*) has only been reported in Algeria and Spain, and no information is available concerning genotypes of *E. bieneusi* in donkeys worldwide. In the present study, a total of 301 donkey fecal samples (48 from Jilin Province, 224 from Shandong Province and 29 from Liaoning Province) were collected and examined by PCR amplification of the internal transcribed spacer (ITS) region. The overall *E*. *bieneusi* prevalence was 5.3% (16/301), with 6.3% (3/48) in Jilin Province, 4.9% (11/224) in Shandong Province, and 6.9% (2/29) in Liaoning Province. Prevalence in different age groups ranged from 4.2 to 5.5%. *E*. *bieneusi* prevalence in donkeys sampled in different seasons varied from 4.2 to 6.5%. Altogether, four *E. bieneusi* genotypes were identified in this study, with two known genotypes (J and D) and two novel genotypes (NCD-1and NCD-2). Phylogenetic analysis revealed that genotypes D, NCD-1 and NCD-2 belonged to group 1, while the remaining genotype J was clustered into group 2. These findings revealed the occurrence of *E. bieneusi* in donkeys in China for the first time. Moreover, the present study also firstly genotyped the *E*. *bieneusi* in donkeys worldwide. These findings extend the distribution of *E. bieneusi* genotypes and provide baseline data for controlling *E. bieneusi* infection in donkeys, other animals and humans.

## Introduction

Microsporidiosis is of increasing concern because Microsporidia can infect virtually all animals (Santín and Fayer, 2011; Abu-Akkada et al., 2015; Santin and Fayer, 2015; Zhang et al., 2016a; Zhao et al., 2016). Fecal-oral routes, such as ingestion of contaminated water and food (Zhao et al., 2014a) are the major route to transmit Microsporidia which consist of 1300 named species (Karim et al., 2014c). Of these, *Enterocytozoon bieneusi* is considered as the most important species and responsible for more than 90% of human microsporidiosis (Desportes et al., 1985). Although *E. bieneusi* was firstly detected in HIV patients in 1985 (Desportes et al., 1985), as research continues, more and more animals were also considered as susceptible hosts for *E. bieneusi*. To date, more than 200 distinct genotypes have been reported on the basis of sequences of the internal transcribed spacer (ITS) region (Karim et al., 2014b; Tian et al., 2015). These genotypes were divided into several groups: the zoonotic groups (syn. Group 1), host-adapted groups (groups 2–5 and an outlier genotypes in dogs), and some other small groups (groups 6–9). However, surprisingly, some of the genotypes in Group 2 (the so-called host-adapted group) were found in both animals and humans, which should also be considered as a zoonotic agent (Hu et al., 2014; Karim et al., 2014a; Ma et al., 2015a,b).

To our knowledge, limited data has been published on genotypes of *E. bieneusi* from China (Tian et al., 2015; Zhang et al., 2015, 2016a,b; Qi et al., 2016) and no data is available for donkeys. The objectives of the present study were to investigate the *E*. *bieneusi* prevalence and identify their genotypes in donkeys in Jilin, Liaoning and Shandong Provinces, eastern and northeastern China.

## Materials and Methods

### Ethics Approval and Consent to Participate

This study was approved by the Animal Ethics Committee of Lanzhou Veterinary Research Institute, Chinese Academy of Agricultural Sciences. Donkeys used for the study were handled in accordance with good animal practices required by the Animal Ethics Procedures and Guidelines of the People’s Republic of China.

### Collection and Preparation of Donkey Fecal Samples

Between May 2015 and October 2016, a total of 301 fecal samples were collected from donkeys from Jilin Province (*n* = 48), Liaoning Province (*n* = 29), and Shandong Province (*n* = 224). At the time of the sampling, all the donkeys were in apparently good health status. Fecal samples were collected from each animal after defecation onto the ground, and then were taken to the laboratory. All the detailed information of investigated donkeys was obtained and listed in **Tables 1**, **2**.

**Table 1:** Factors associated with prevalence of *Enterocytozoon bieneusi* in donkeys in Northern China.

Factor	Category	No. tested	No. positive	% (95% CI)	*P*-value	OR (95% CI)
Region	Shandong Province	224	11	4.9 (2.1-7.7)	0.86	Reference
	Liaoning Province	29	2	6.9 (0.0 - 16.1)		1.4 (0.3 - 6.8)
	Jilin Province	48	3	6.3 (0.0 - 13.1)		1.3 (0.4 - 4.8)
Age	Young	48	2	4.2 (0.0 - 9.8)	0.70	Reference
	Adult	253	14	5.5 (2.7 - 8.4)		1.4 (0.3 - 6.1)
Season	Winter	104	6	5.8 (1.3 - 10.3)	0.75	Reference
	Summer	77	5	6.5 (1.0 - 12.0)		1.1 (0.3 - 3.9)
	Autumn	120	5	4.2 (0.6 - 7.7)		0.7 (0.2 - 2.4)
Total		301	16	5.3 (2.8 - 7.9)		

**Table 2:** *Enterocytozoon bieneusi* genotypes identified in donkeys in different farms.

Region	Farm ID	Age category (*n*)	No. positive/no. tested (%)	Genotype (*n*)
Jilin Province	Farm 1	Young (10), Adult (38)	3/48 (6.3)	D (*n* = 2), NCD-1 (*n* = 1)
Liaoning Province	Farm 2	Young (6), Adult (23)	2/29 (6.9)	D (*n* = 1), J (*n* = 1)
Shandong Province	Farm 3	Young (14), Adult (35)	3/49 (6.1)	J (*n* = 3)
	Farm 4	Young (18), Adult (37)	3/55 (5.5)	D (*n* = 1), J (*n* = 2)
	Farm 5	Adult (120)	5/120 (4.2)	J (*n* = 4), NCD-2 (*n* = 1)
Total		Young (48), Adult (253)	16/301 (5.3)	D (*n* = 4), J (*n* = 10), NCD-1 (*n* = 1), NCD-2 (*n* = 1)

### DNA Extraction and PCR Amplification

Genomic DNA from each fecal sample was obtained by using a DNA extraction kit (OMEGA, USA). The extracted DNA samples were stored at -20°C until PCR amplification.

The *E. bieneusi* genotypes were determined by the nested PCR of the ITS region of the ribosomal RNA (rRNA) gene using primers F1 [5′-GGTCATAGGGATGAAGAG-3′] and R1 [5′-TTCGAGTTCTTTCGCGCTC-3′] for primary amplification, and primers F2 [5′-GCTCTGAATATCTATGGCT-3′] and R2 [5′-ATCGCCGACGGATCCAAGTG-3′] for secondary amplification. 25 μl of PCR reaction composed of 200 μM each deoxy-ribonucleoside triphosphate (dNTP), 1 × Ex *Taq* buffer (Mg^2+^ free), 0.4 μM of each primer, 0.625 U of Ex *Taq* DNA polymerase (TAKARA, Japan), 2 mM MgCl_2_, and 2 μl of DNA template. Both positive and negative controls were included in each test. All the PCR products were detected under UV light after electrophoresis on a 2% agarose gels containing GoldView^TM^ (Solarbio, China).

### Sequence and Phylogenetic Analyses

Positive secondary PCR products were sequenced using bi-directional sequencing. Genotypes that produced sequences with mutations, including single nucleotide substitutions, deletions or insertion which were confirmed by the DNA sequencing of at least two PCR products, were identified as novel genotypes. The obtained sequences were aligned with reference sequences to determine the *E*. *bieneusi* genotypes/subtypes using the BLAST^1^. Phylogenetic trees were reconstructed using neighbor-joining (NJ) method in Mega 5.0 software (Kimura 2-parameter model, 1000 replicates).

### Statistical Analysis

The variation in *E*. *bieneusi* prevalence (*y*) of donkeys of different geographical location (*x*1), season (*x*2) and age (*x*3) were analyzed by χ^2^ test using SPSS V20.0 (IBM, Chicago, IL, USA) (Letchumanan et al., 2015; Cabal et al., 2016). Using multivariable regression analysis, each of these variables was included in the binary logit model as an independent variable. The best model was judged by Fisher’s scoring algorithm. All tests were two-sided. When the values of *P* < 0.05, results were considered statistically significant. 95% confidence intervals (95% CIs) were also calculated based on the formula of *X* ± *Z* × {*X*(1 - *X*) / *N*}^½^ (*Z* = 1.96; *X* represents the prevalence; *N* represents the sample sizes).

## Results and Discussion

A total of 16 (5.3%) out of 301 donkeys were PCR-positive for *E. bieneusi* (**Table 1**). Donkeys in Shandong Province (11/224, 4.9%) showed the higher *E. bieneusi* prevalence (**Table 1**). *E. bieneusi* prevalence in different season groups ranged from 4.2 to 6.5% (**Table 1**). Prevalence of *E. bieneusi* in young donkeys and adult donkeys was 4.2 and 5.5%, respectively (**Table 1**). Moreover, *E. bieneusi* prevalence in different farm groups varied from 4.2 to 6.9% (**Table 2**).

In this study, the overall prevalence was 5.3% (16/301) which was lower than that in horses from the Czech Republic (6.9%) (Wagnerová et al., 2012), Colombia (10.8%) (Santín et al., 2010), and Algeria (6.8%). However, it is higher than that in donkeys in Algeria (1.6%) (Laatamna et al., 2015), and Spain (0%) (Lores et al., 2002). Moreover, it is also considerably higher than that in horses in Spain (0%) (Lores et al., 2002). The different infection rates may be due to the different susceptibility between donkeys and horses. Moreover, animal welfare, sample sizes, age distribution of the samples and geo-ecological conditions can also influence the *E. bieneusi* prevalence.

In the present study, although donkeys from Liaoning Province has relatively high rates of *E*. *bieneusi* infection compared with donkeys from Jilin Province and Shandong Province, the difference was not statistically significant (χ^2^ = 0.30, *df* = 2, *P* = 0.86) (**Table 1**). This may be due to the similar climates during sampling times in Jilin, Liaoning and Shandong Provinces, and also relate to the same management mode in these farms. Moreover, the present study also showed that *E*. *bieneusi* prevalence in donkeys increased gradually with age, which is consistent with previous reports in that *E. bieneusi* may accumulate throughout the life time (Thellier and Breton, 2008; Zhao et al., 2014a), but the difference was not statistically significant (χ^2^ = 0.15, *df* = 1, *P* = 0.70) (**Table 1**).

**FIGURE 1:**
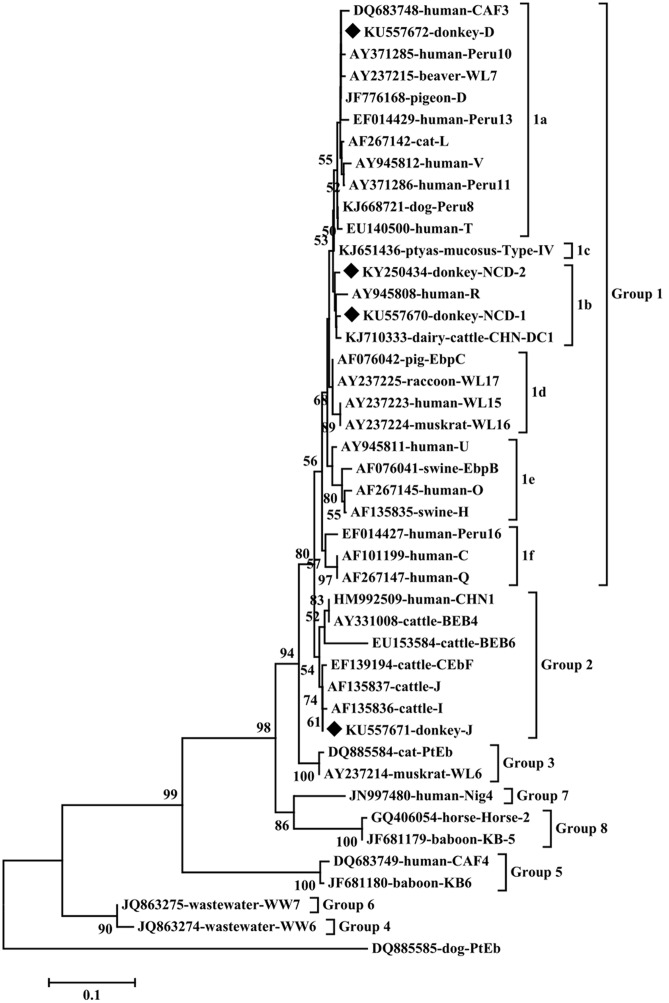
**Phylogenetic analyses of *Enterocytozoon bieneusi* using neighbor-joining (NJ) method (Kimura 2-parameter model)**. Bootstrapping (1000 replicates) was performed. The values below 50% are not shown. *E. bieneusi* isolates identified in the present study are indicated by solid diamond.

To our knowledge, although two studies of *E. bieneusi* prevalence in donkeys have been reported previously, these isolates were not genotyped successfully (Lores et al., 2002; Laatamna et al., 2015). Previous studies have identified sixteen *E. bieneusi* ITS genotypes, namely Horse 1 – Horse 11, G, WL15, D, EpbA and CZ3 in horses who is a close relative to donkeys (Santín et al., 2010; Wagnerová et al., 2012; Laatamna et al., 2015). But probably due to the smaller sample sizes and different collection times, only four *E. bieneusi* genotypes were observed in donkeys in the present study, including two known genotypes J and D, and two novel genotypes NCD-1 and NCD-2 (**Figure 1**). Thus, four *E. bieneusi* genotypes were endemic in donkeys in eastern and northeastern China. This study also indicated that genotype J was the most prevalent in donkeys, which were different from the results reported in horses in Colombia (Santín et al., 2010) and Czech Republic (Wagnerová et al., 2012), where the predominance of genotype D was found, and in horses in Algeria, where predominance of horse1 was identified (Laatamna et al., 2015). Furthermore, a total of 10 polymorphic sites were observed among these genotypes (**Table 3**), which suggested the high genetic diversity of *E. bieneusi* in the investigated donkeys.

**Table 3:** Variations in the ITS nucleotide sequences among genotypes of the *Enterocytozoon bieneusi* in donkeys in Northern China.

Genotypes (no.)	Nucleotide at position										GenBank accession nos.
	107	157	169	189	193	205	207	213	223	234	
J (Reference)	G	T	T	T	G	A	C	T	A	A	KP735178
J (*n* = 10)	G	T	T	T	G	A	C	T	A	A	KU557671
D (*n* = 4)	G	C	C	C	T	G	G	C	G	T	KU557672
NCD-1 (*n* = 1)	G	C	T	C	G	G	A	C	A	T	KU557670
NCD- 2 (*n* = 1)	A	C	T	C	G	G	G	C	A	T	KY250434

Genotypes J and D have been widely identified in different hosts in northern and eastern China. For example, genotype D was identified in golden takins (*Budorcas taxicolor bedfordi*) in Shannxi (Karim et al., 2014a), non-human primates in Henan (Wang et al., 2013; Karim et al., 2014b), dairy cattle, sheep, goats, pig, raccoon dog, cats, blue foxes and dogs in Heilongjiang (Zhao et al., 2014a,b, 2015a,b; Li et al., 2015), and blue foxes in Jilin (Zhao et al., 2015c); genotype J was found in cattle in Heilongjiang, Jilin, Shandong, Henan, captive wildlife in Henan, yaks in Qinghai. In addition, genotypes D was also identified in HIV patients in Henan (Wang et al., 2013; Karim et al., 2014b) and J was found in a child in Jilin (Zhang et al., 2011). Moreover, two of the ITS sequences (accession numbers: KU557671 and KU557672) of the identified *E. bieneusi* isolates were identical to that of genotypes J (GenBank accession no. KP735178, from a *Homo sapiens* in Iran) and D (GenBank accession no. KP262379, from a goat in China) sequences available in GenBank, respectively. These results indicate cross-species transmission of these *E. bieneusi* genotypes in northern China.

Phylogenetic analysis indicated that genotypes D, NCD-1 and NCD-2 belonged to group 1, the most important zoonotic groups; J was grouped into group 2, a cattle-specific groups, but genotype J has also been identified in humans. These results suggest that donkeys are potential source of animal and human microsporidiosis.

## Conclusion

This is the first study of *E*. *bieneusi* prevalence (5.3%, 16/301) in donkeys in China. Moreover, two known genotypes (genotypes D and J), and two novel genotypes (NCD-1 and NCD-2) were detected for the first time in donkeys. Donkeys should be considered as an important potential source of human microsporidiosis, and effective strategies should be performed to control *E*. *bieneusi* infection in donkeys, other animals and humans.

## Data Availability Statement

Representative nucleotide sequences were deposited in GenBank with the following accession numbers: KU557670-KU557672 and KY250434.

## Author Contributions

X-QZ and X-XZ conceived and designed the study, and critically revised the manuscript. D-MY, J-GM, and W-BZ performed the experiments. X-XZ and D-MY analyzed the data. D-MY and J-GM drafted the manuscript. F-CL, J-LH and QZ helped in study design, study implementation and manuscript preparation. All authors read and approved the final manuscript.

## Conflict of Interest Statement

The authors declare that the research was conducted in the absence of any commercial or financial relationships that could be construed as a potential conflict of interest.

## Footnotes

1 http://www.ncbi.nlm.nih.gov/BLAST/
